# Dysmenorreœa

**Published:** 1854-02

**Authors:** 


DYSMENORRHŒA.—In Germany the bi-borate of soda is used in conjunction with ergot, to produce uterine contraction, and alone to facilitate the excretion of the menstrual fluid. Dr. Bennett, of London, regards it as an important agent in fulfilling the latter indication, and employs the following formula in many cases of dysmenorrhœa:—℞. magnes. carbon., gr. v.; pulveris rhei., gr. ij.; sodæ biboratis, gr. x.; infusi columb., ℥j.; *ter die summend.*—*Med. Times and Gazette.*

